# QuinoxalineTacrine QT78, a Cholinesterase Inhibitor as a Potential Ligand for Alzheimer’s Disease Therapy

**DOI:** 10.3390/molecules24081503

**Published:** 2019-04-17

**Authors:** Eva Ramos, Alejandra Palomino-Antolín, Manuela Bartolini, Isabel Iriepa, Ignacio Moraleda, Daniel Diez-Iriepa, Abdelouahid Samadi, Carol V. Cortina, Mourad Chioua, Javier Egea, Alejandro Romero, José Marco-Contelles

**Affiliations:** 1 Department of Pharmacology and Toxicology, Faculty of Veterinary Medicine, Complutense University of Madrid, 28040 Madrid, Spain; eva.ramos@ucm.es; 2 Molecular Neuroinflammation and Neuronal Plasticity Laboratory, Research Unit, Hospital Universitario Santa Cristina, 28009 Madrid, Spain; apantolin@gmail.com; 3 Instituto de Investigación Sanitaria, Hospital Universitario de la Princesa, 28006 Madrid, Spain; 4 Instituto-Fundación Teófilo Hernando, Departamento de Farmacología y Terapéutica, Universidad Autónoma de Madrid, 28029 Madrid, Spain; 5 Department of Pharmacy and Biotechnology, Alma Mater Studiorum University of Bologna, Via Belmeloro 6, 40126 Bologna, Italy; manuela.bartolini3@unibo.it; 6 Departamento de Química Orgánica and Química Inorgánica. Ctra. Madrid-Barcelona, Km. 33,6. Universidad de Alcalá, 28871 Madrid, Spain; isabel.iriepa@uah.es (I.I.); ignacio.moraleda@uah.es (I.M.); daniel.diezi@edu.uah.es (D.D.-I.); 7 Laboratory of Medicinal Chemistry (IQOG, CSIC), C/Juan de la Cierva 3, 28006 Madrid, Spain; samadi@uaeu.ac.ae (A.S.); cvcortina@gmail.com (C.V.C.); mchioua@gmail.com (M.C.); 8 Department of Chemistry, College of Science, United Arab Emirates University, 15551 Al Ain, UAE

**Keywords:** Alzheimer’s disease, cholinesterase inhibitor, hepatotoxicity, molecular modeling, neuroprotection, quinoxalines, quinoxalinetacrines, tacrine

## Abstract

We report the synthesis and relevant pharmacological properties of the quinoxalinetacrine (QT) hybrid **QT78** in a project targeted to identify new non-hepatotoxic tacrine derivatives for Alzheimer’s disease therapy. We have found that **QT78** is less toxic than tacrine at high concentrations (from 100 μM to 1 mM), less potent than tacrine as a ChE inhibitor, but shows selective BuChE inhibition (IC_50_ (hAChE) = 22.0 ± 1.3 μM; IC_50_ (hBuChE) = 6.79 ± 0.33 μM). Moreover, **QT78** showed effective and strong neuroprotection against diverse toxic stimuli, such as rotenone plus oligomycin-A or okadaic acid, of biological significance for Alzheimer’s disease.

## 1. Introduction

Alzheimer’s disease (AD) is the most common neurodegenerative disorder. Many biochemical hallmarks are involved in the progress and development of this pathology: extracellular senile plaques that appear by amyloid-beta peptide deposition and aggregation, hyperphosphorylated tau-protein, neuroinflammation, cell death, oxidative stress, and a strong deficit of neurotransmitters such as acetylcholine, dopamine, and serotonin [1]. These facts have resulted in what has been called the “cholinergic hypothesis of AD”, highlighting that most AD patient impairments are due to deficient cholinergic neurotransmission [2]. Consequently, a few acetylcholinesterase (AChE) and butyrylcholinesterase (BuChE) inhibitors (rivastigmine, donepezil, galantamine) have been identified and are currently being used in clinical treatment for the therapy of AD [3]. The finding that BuChE activity is raised in AD, and that BuChE activity is concentrated in areas of the brain engaged in cognition, suggests the use of selective BuChE inhibitors for AD therapy [4]. Unfortunately, to date, no effective treatment has been discovered to cure this pathology. Consequently, there is an urgent need for novel and more efficient drugs to treat AD patients. In the search for new therapies to deal with this complex and multifactorial disease, the multitarget small molecule (MTSM) approach [5] is considered one of the most attractive therapeutic strategies. 

**Figure 1:**
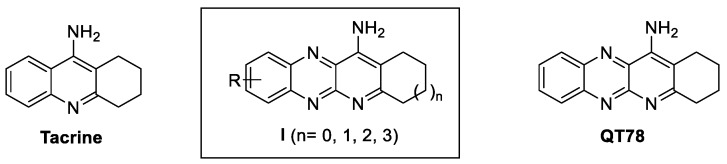
General structure for quinoxalinetacrines (QTs) (I), tacrine, and **QT78**.

In this context, and pursuing our search for non-hepatotoxic tacrine (Figure 1) analogues [6] showing ChE inhibition, we designed the quinoxalinetacrines (QTs) (**I**, Figure 1) as new, simple, and readily available agents. Quinoxalines are a class of molecules endowed with a large of array of biological properties, including anti-neuroinflammation, antitumoral, antimycobacterial, and antifungal activities [7]. 

In this article, we describe the synthesis, toxicological and neuroprotective properties, as well as the computational analysis of **QT78** (1,2,3,4-tetrahydroquinolino[2,3-*b*]quinoxalin-12-amine) (Figure 1), as the first member of this new family of QT derivatives.

## 2. Results and Discussion

**Scheme 1:**
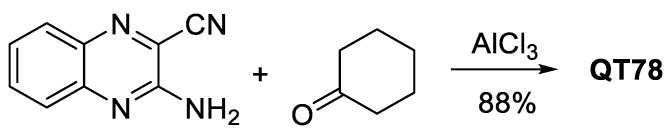
Synthesis of QT78.

The synthesis of **QT78** has been carried out as shown in Scheme 1, with a good yield, by reacting 3-amino-2-quinoxalinecarbonitrile [8] and cyclohexanone, under typical Friedländer-type reaction conditions [9,10]. **QT78** showed spectroscopic data in good agreement with its structure and excellent analytical data (see Supporting Information). It is interesting to note that Hamama and co-workers have recently reported the reaction of 2-amino-3-cyano-6-methylquinoxaline-1,4-dioxide with several cycloalkanones, and described their antibacterial, antifungal, and antitumor activities [11].

**Figure 2:**
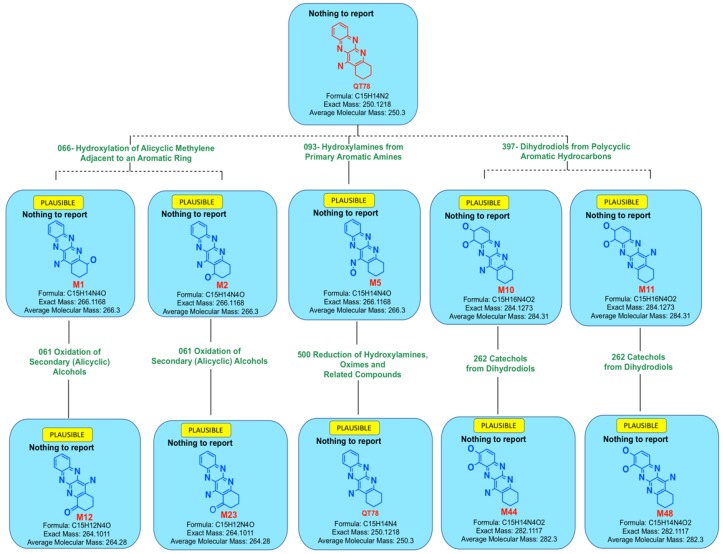
Summary metabolic tree after processing **QT78** (query compound) for human Phase I biotransformation reactions and two sequences of biotransformation.

First, we started with the in silico toxicological analysis [12]. In order to predict the hepatotoxic potential of **QT78**, and its main Phase I metabolites, we performed the in silico analysis of **QT78**. The result for hepatotoxicity was “nothing to report”, showing that there is not enough evidence to predict a positive/negative result. This means that, structurally, the novel compound is quite different to tacrine, and consequently, **QT78** does not provide any positive prediction of hepatotoxicity. Likewise, its ten Phase I metabolites displayed the same message, indicating that there was no associated tacrine metabolites risk (Figure 2).

Thus, we chose HepG2 cells, a well-established in vitro model and widely used human hepatoma cell line [13], to test the hepatotoxicity exerted by QT78, using tacrine as the reference drug. Cells were incubated with **QT78** and tacrine in a wide range of doses (1–1000 μM) in a 24 h period. The HepG2 viability was significantly decreased when **QT78** was incubated at the highest concentrations (300 and 1000 μM) (Table 1). However, the toxicity profile was significantly better than that of tacrine, which showed a significant viability reduction at lower concentrations (from 30 μM). These results suggest that the hepatotoxic safety profile of **QT78** is better than that of tacrine (Supporting Information).

Next, the in vitro inhibition of human acetylcholinesterase (hAChE) and human butyrylcholinesterase (hBuChE) was assessed according to the method of Ellman [14] (Supporting Information), using tacrine as the reference compound. Comparison of the IC_50_ values highlighted that **QT78** acts as a modest but selective hBuChE inhibitor [IC_50_ (hAChE) = 22.0 ± 1.3 μM; IC_50_ (hBuChE) = 6.79 ± 0.33 μM]. Thus, it is obvious that the enlargement of the three-membered tetrahydroacridine scaffold to the four-membered tetrahydroquinolino[2,3-*b*] quinoxaline nucleus was detrimental regarding its activity towards both ChE enzymes, leading to a decrease of the inhibitory potency of two orders of magnitude (tacrine: IC_50_ (hAChE) = 0.374 ± 0.053 μM; IC_50_ (hBuChE) = 0.044 ± 0.002 μM) [10]. Based on these results, we next carried out docking studies to explain the binding modes of compound **QT78** in hAChE and hBuChE. 

The structures of hAChE crystallized with fasciculin II (PDB ID: 1B41) and hBuChE crystallized with tacrine (PDB ID: 4BDS) were used. The docking of compound **QT78** with the target enzymes was performed using AutoDock Vina [15] software and the results were analyzed with Discovery Studio. As we have previously described [10], the conformational flexibility of AChE was considered by allowing eight side chains to be flexible during the docking process.

**Figure 3:**
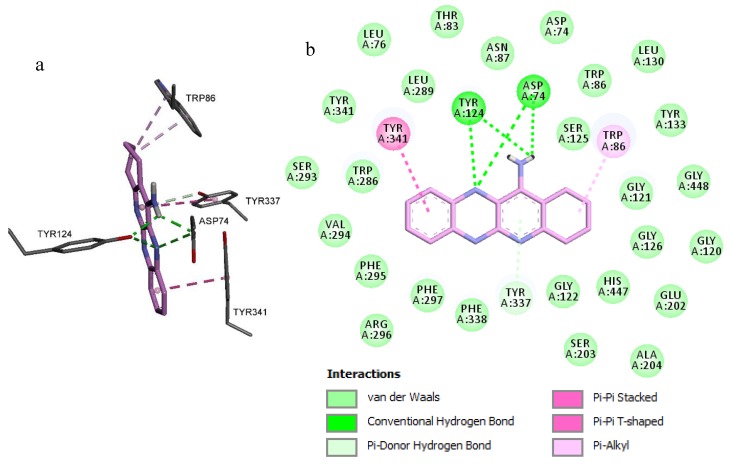
(**a**) Three-dimensional representation of interactions established by **QT78** with hAChE. (**b**) Two-dimensional schematic view of the interactions between **QT78** and hAChE.

Regarding hAChE, compound **QT78** is accommodated in the middle of the active-site gorge and no interaction with the catalytic triad aminoacids (His447, Ser203 and Glu334) is established. The cyclohexane ring is oriented towards the bottom of the gorge, establishing π-alkyl interactions with Trp86, a residue involved in the interaction with the quaternary amine of the natural ligand ACh. The complex AChE-**QT78** was also stabilized by molecular interactions with several key residues at the peripheral anionic site (PAS). There is a hydrogen bond network in which the amino group and the pyrazine nitrogen with Tyr124 and Asp74 are involved. Additionally, the phenyl ring forms a π-π stacking interaction with Tyr341 and the pyridine ring forms a π-π T-shaped interaction with Tyr337 (Figure 3a,b). To sum up, we propose that compound **QT78** interacts with the PAS of AChE without catalytic triad residue involvement.

**Figure 4:**
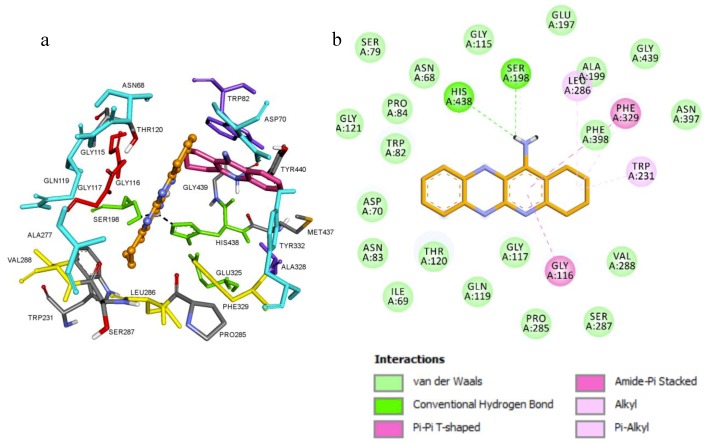
(**a**) Molecular docking model for compound **QT78** and tacrine within the hBuChE binding-site. Compound **QT78** is colored orange, and tacrine, in cherry. Different subsites of the active site were colored: catalytic anionic site (CAS) in green, oxyanion hole (OH) in red, choline binding site in violet (CBS), acyl binding pocket (ABP) in yellow, and peripheral anionic site (PAS) in blue. Hydrogen bonding interactions are shown in dashed black lines. (**b**) Two-dimensional interaction map of compound **QT78**, which is bound to the active site of hBuChE.

Docking within hBuChE shows that compound **QT78** is deeply inserted into the active site. Nevertheless, it can be seen that the structure of the docked compound does not fit in the space occupied by tacrine (Figure 4a), which could account for its weaker BuChE inhibitory activity. As shown in Figure 4b, the binding mode of compound **QT78** is characterized by the following features: the NH_2_ group forms two hydrogen bonds with the catalytic triad residues His438 and Ser198. Moreover, the pyridine and cyclohexane rings interact with the residues in the acyl-binding pocket (ABP) (Phe329 and Leu286). Also, the pyridine ring allowed an amide-π stacked interaction with Gly116 in the oxyanion hole (OH).

The molecular docking results show that **QT78** is located in the active site of both enzymes, but in hBuChE, the ligand interacts with the catalytic triad residues, whereas in hAChE the ligand is further away. These results agree with the experimental results, which showed that QT78 is a modest but selective hBuChE inhibitor.

In addition, for essential biological activity, drug candidates should also have an ideal pharmacokinetic profile. In this context, we have computed Lipinski’s rule of five and other pharmacokinetic properties of **QT78**, using the QikProp module of Schrödinger software (QikProp, version 3.8, Schrodinger, LLC, New York, NY, USA, 2013).

Compounds violating more than one of these rules may have bioavailability limitations. The ADME predictions of compound **QT78** showed satisfactory results highlighting drug-like characteristics based on Lipinski’s rule of five (see Table S1, Supporting Information) [16]. 

The drugs used for neurological disorder treatment act on the central nervous system (CNS). CNS drugs show values of MW < 450, hydrogen-bond donors < 3, hydrogen-bond acceptors < 7, number of hydrogen bond donor < 5, van der Waals surface area of polar nitrogen and oxygen atoms < 90, number of rotatable bonds < 8 and hydrogen bonds < 8. **QT78** satisfied all the characteristic of CNS acting drugs. 

The aqueous solubility (QPlogS) of organic compounds plays a key impact on many ADME-related properties. The solubility value of **QT78** (QPlogS = −3.746) was within the limits (−6.5–0.5). Further, the predicted value for the partition coefficient (QPlogPo/w = 2.534), critical for the estimation of absorption within the body, is in the optimum range (−2.0–6.5). 

Drugs must cross the blood–brain barrier (BBB) to exert their activity on the CNS; thus, one of the most used descriptors for CNS penetration is logBB. Experimental values of log BB cover a span from about −2.00 to +1.04. Within this range, compounds with log BB > 0.30 cross BBB readily, while compounds with a log BB < −1.00 are poorly distributed into the brain [17]. The QPlogBB value for **QT78** is −0.332, indicating it has a good potential for BBB penetration. 

The literature data suggest that polar surface area (PSA) is a measure of the hydrogen bonding capacity of a molecule, and its value should not exceed a certain limit if the compound is intended to be CNS active. The most active CNS drugs have a PSA lower than 70 A^2^. The value of PSA for **QT78** is 59.549 Å^2^, suggesting a good penetration of the BBB. Similarly, the percentage of human oral absorption is very high (100%), while other physicochemical descriptors obtained by QikProp (Table S1, Supporting Information) are within the acceptable range for human use, thereby indicating that **QT78** is a potential drug-like molecule and possible CNS drug.

**Figure 5:**
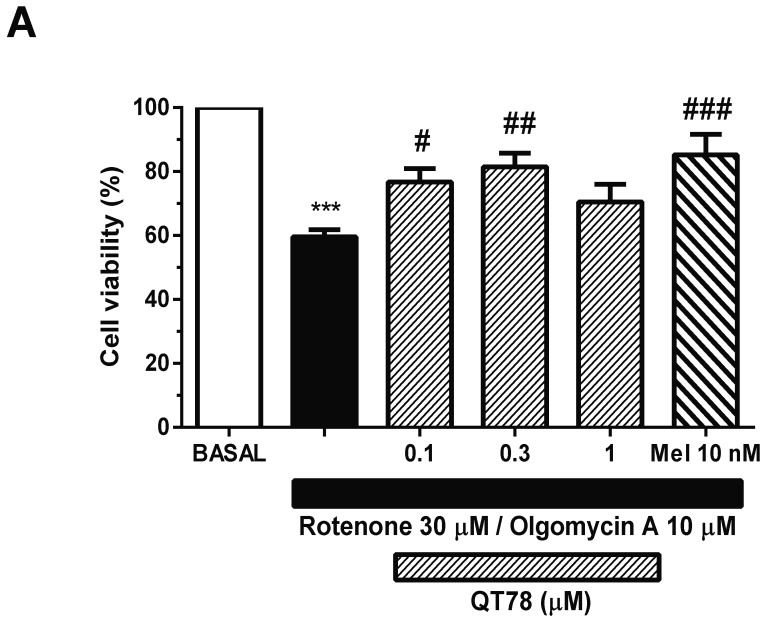

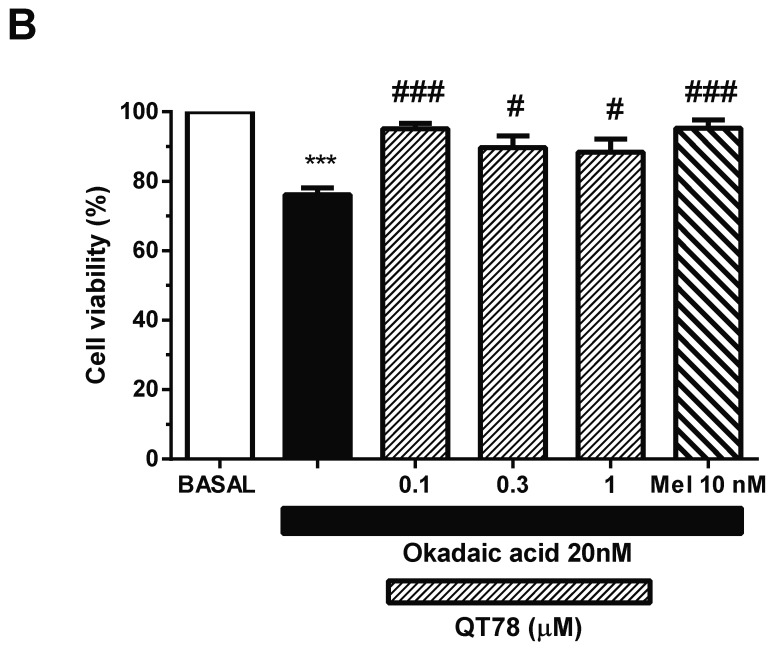
Neuroprotective properties of **QT78** (0.1, 0.3 and 1 μM) in SH-SY5Y cells against (**A**) rotenone (30 μM)/oligomycin A (10 μM) or (**B**) okadaic acid (20 nM) toxicity. Data are expressed as means ± SEM of five different cultures performed in triplicate (untreated control set to 100%). *** *p* < 0.001, compared to control; ^###^ *p* < 0.001, ^##^ *p* < 0.01 and ^#^ *p* < 0.05 compared to toxic stimuli.

Finally, following the protocols described in the Supporting Information, we evaluated the neuroprotective profile of **QT78** against two toxic insults related to neurodegeneration [18,19], using melatonin (10 nM) as a reference compound: (a) a model of reactive oxygen species (ROS) generation, based on the cocktail of the mitochondrial respiratory chain blockers rotenone and oligomycin A (R/O), and (b) hyperphosphorylation of tau protein using okadaic acid (OA), a well-known protein phosphatase inhibitor. As shown in Figure 5, **QT78** revealed an interesting neuroprotective activity. At the concentrations of 0.1 and 0.3 μM, **QT78** produced a significant increase in cell viability against R/O (16.7% and 21.4%, respectively). Similarly, **QT78** at 0.1 μM showed a potent increase in cell viability against OA (18.9%). In all cases, when we increased the concentration of the compound to 1 μM, the protective capacity decreased against all toxic stimuli tested.

## 3. Materials and Methods

### 3.1. Chemistry Methods

Reactions were monitored by TLC using precoated silica gel aluminum plates containing a fluorescent indicator (Merck, 5539, Kenilworth, NJ, United States). Detection was done by UV (254 nm) followed by charring with sulfuric–acetic acid spray, 1% aqueous potassium permanganate solution or 0.5% phosphomolybdic acid in 95% EtOH. Anhydrous Na_2_SO_4_ was used to dry organic solutions during work-ups and the removal of solvents was carried out under vacuum with a rotary evaporator. Flash column chromatography was performed using silica gel 60 (230–400 mesh, Merck). Melting points were determined on a Kofler block and are uncorrected. IR spectra were obtained on a Perkin-Elmer Spectrum One spectrophotometer (Waltham, MA, United States). ^1^H-NMR spectra were recorded with a Varian VXR-200S spectrometer (Palo Alto, CA, United States), using tetramethylsilane as internal standard and ^13^C-NMR spectra were recorded with a Bruker WP-200-SY (Billerica, MA, United States). All the assignments for protons and carbons were in agreement with 2D COSY, HSQC, HMBC, and 1D NOESY spectra. Values (*) can be interchanged. The purity of compounds was checked by elemental analyses, conducted on a Carlo Erba EA 1108 apparatus (Sabadell, Spain), and confirmed to be >95%. 

### 3.2. Synthesis of **QT78**

**Figure 6:**
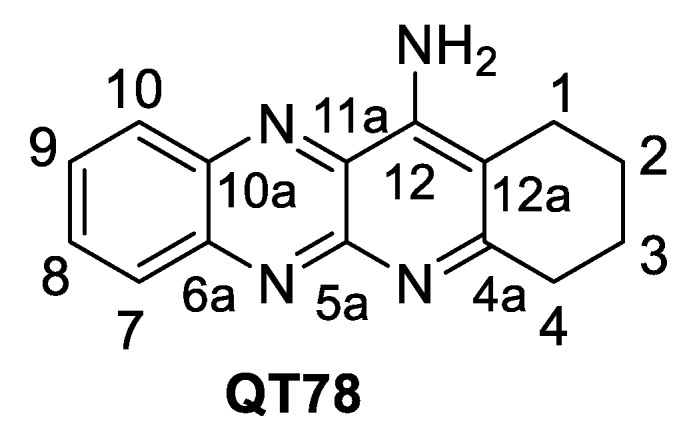
Structure of **QT78**.

A mixture of 3-amino-2-quinoxalinecarbonitrile [8] (160 mg, 0.94 mmol), cyclohexanone (146 µL, 1.41 mmol) and AlCl_3_ (186 mg, 1.41 mmol) in 1,2-dichloroethane (6 mL) was irradiated in a MW apparatus at 95 °C, 250 W and 20 atm, for 2 h. Then, the mixture was cooled at 0 °C, diluted with a mixture of H_2_O/THF (1:1), treated with NaOH 15% until basic, and extracted several times with methylene chloride. The organic layer was washed with brine until neutral, dried with Na_2_SO_4_, filtered and evaporated. The residue was purified by column chromatography (CH_2_Cl_2_/MeOH (from 1% to 5%)), affording 1,2,3,4-tetrahydroquinolino[2,3-*b*]quinoxalin-12-amine (**QT78,** Figure 6) (172 mg, 88%): mp 238–240 °C; IR (KBr) 3292, 2938, 1622, 1605, 1501, 1375, 1347, 1326, cm^−1^; ^1^H-NMR (300 MHz, DMSO) δ 8.17 (dd, *J*= 2.1, 8.8 Hz, 2H, H7, H10), 8.01–7.70 (m, 2H, H8, H9), 7.10 (bs, 2H, NH_2_), 2.98–2.96 (m, 2H, H4), 2.69–2.56 (m, 2H, H1), 1.88–1.85 (m, 4H, H2, H3); ^13^C-NMR (75 MHz, DMSO) δ 165.8 (2C, C12, C4a), 148.9 (C5a), 147.5 (C11a), 144.2 (C10a)*, 140.0 (C6a)*, 131.0 (C9)**, 130.6 (C8)**, 129.3 (C7)***, 129.0 (C10)***, 110.3 (C12a), 34.5 (C4), 23.9 (C1), 22.3 (C2)*, 21.9 (C3)*; MS (EI) *m/z* (%): 250 [M^+^] (100), 235 (18). HRMS. Calc. for C_15_H_14_N_4_ (M + H)^+^: 251.12912. Found: 251.12989. Anal. Cald. for C_15_H_14_N_4_: C, 71.98; H, 5.64; N, 22.38. Found: C, 71.75; H, 5.61; N, 22.15.

### 3.3. In Silico Prediction Systems for Toxicology and Metabolism

Meteor Nexus (v.3.1.0, Lhassa Limited, Leeds, UK) and Derek Nexus (v.6.0.1, Lhassa Limited, Leeds, UK) knowledge-based expert systems (Knowledge Bases: Meteor KB 2015 1.0.0, Derek KB 2015 1.0) were employed for metabolism and toxicity predictions [20,21]. Hepatotoxicity was selected as an “end point”. Derek Nexus assesses predictions based on a summary of evidences, evaluating alerts and estimating the likelihood of occurrence [21]. The compound was considered to have a structural alert for the selected endpoint (hepatotoxicity) if the prediction in Derek Nexus™ was ‘certain’, ‘probable’, ‘plausible’ or ‘equivocal’. The predictions ‘doubted’, ‘improbable’, ‘impossible’, ‘inactive’ or ‘no alert’ were regarded as negative. When Derek has no knowledge on which to base a prediction, the message “nothing to report” is displayed; this absence of evidence is not synonymous with a prediction of inactivity.

The metabolism was predicted by Meteor Nexus version 3.1.0, (Lhasa ltd., Leeds, UK), a SAR (structure-activity relationship) tool used to predict the likely metabolic fate of a chemical structure through a knowledge-base composed of a biotransformation dictionary, rules and example metabolic pathways. Meteor was setup for human Phase I biotransformation reactions, since in most cases the reactive metabolites are generated particularly through Phase I metabolic reactions [22]. An absolute reasoning biotransformation ranking method was applied as a qualitative rule-based approach to evaluate the likelihood level (probable, plausible, equivocal, doubted, and improbable). The minimal likelihood level selected was “plausible”, meaning that the weight of evidence supports the proposition. All possible first and second-generation metabolites have been generated and afterwards processed for hepatotoxicity predictions. 

### 3.4. In Vitro Toxicity of **QT78** and Tacrine in HepG2 Cells

The human hepatoma HepG2 cell line was kindly provided by the IdiPAZ Institute for Health Research (Madrid, Spain). They were cultured in Eagle´s minimum essential medium (EMEM) supplemented with 15 nonessential amino acids, 1 mM sodium pyruvate, 10 % heat-inactivated FBS, 100 units/mL penicillin, and 100 µg/mL streptomycin (reagents from Invitrogen, Madrid, Spain). Cultures were seeded into flasks containing supplemented medium and maintained at 37 °C in a humidified atmosphere of 5% CO_2_ and 95% air. Culture media were changed every 2 days. Cells were sub-cultured after partial digestion with 0.25% trypsin-EDTA. For assays, cells were subcultured in 96-well plates at a seeding density of 1 × 10^5^ cells per well. When the cells reached 80% confluence, the medium was replaced with fresh medium containing 1–1000 µM **QT78** and tacrine, or 0.1% DMSO as a vehicle control.

### 3.5. Inhibition of Human AChE and BuChE

The ability of **QT78** to inhibit human ChE activity was assessed using Ellman’s assay [14]. A Jasco V-530 double beam spectrophotometer connected to a HAAKE DC30 thermostating system (Thermo Haake, Vreden, Germany) was used. Stock solutions of the tested compound and reference compound tacrine (1–2 mM) were prepared in methanol and water, respectively, and diluted in the same solvent used to prepare stock solution. The assay solution consisted of a 0.1 M phosphate buffer, pH 8.0, with the addition of 340 μM 5,5′-dithiobis (2-nitrobenzoic acid), 0.02 unit/mL human recombinant AChE or BuChE from human serum (Sigma-Aldrich, Milan, Italy), and 550 μM substrate, i.e., acetylthiocholine iodide or butyrylthiocholine iodide, respectively (Sigma-Aldrich, Milan, Italy). Either 50 µL of a solution of the tested compound at increasing concentrations or 50 µL of solvent (methanol or water) were added to the assay solution and preincubated at 37 °C with the enzyme for 20 min before the addition of substrate. The rate of absorbance increase at 412 nm was followed for 3 min. In parallel, blanks containing all components except the enzyme were prepared to account for the non-enzymatic hydrolysis of the substrate. The reaction rates were compared and the percentage of inhibition due to the presence of test compound was calculated. Each concentration was analyzed in duplicate/triplicate. The percentage of inhibition of the enzyme activity due to the presence of inhibitor was calculated. Inhibition plots were obtained for each compound by plotting the percentage of inhibition versus the logarithm of inhibitor concentration in the assay solution. The linear regression parameters were determined for each curve and the IC_50_ extrapolated.

### 3.6. Molecular Modeling

The structure of compound **QT78** was built using Discovery Studio 2.1 [15]. The molecular geometry of **QT78** was energy-minimized using the adopted-based Newton–Raphson algorithm with the CHARMm force field [23] with a convergence criterion for the energy gradient 0.01 kcal (mol-Å)^−1^. The ligand was set up for docking with the help of AutoDockTools (ADT; version 1.5.4) to define the torsional degrees of freedom to be considered during the docking process. All the rotatable bonds of the ligand were allowed to rotate freely. The enzyme structure used for the calculations was the human AChE (Protein Data Band code 1B41). To give flexibility to the binding site, residues lining the site were allowed to move. These residues were Trp286, Tyr124, Tyr337, Tyr341, Tyr72, Asp74, Thr75, Trp86. Docking calculations for hBuChE were performed following a similar protocol to that described before for hAChE. The coordinates of hBuChE were obtained from the Protein Data Bank (PDB ID: 4BDS). For docking purposes, initial proteins were prepared by removing all water molecules, heteroatoms, any co-crystallized solvent and the ligand. Proper bonds, bond orders, hybridization and charges were assigned using protein model tool in the Discovery Studio, version 2.1, software package. A CHARMm force field was applied using the receptor ligand interactions tool in the Discovery Studio software package. ADT was used to add hydrogens and partial charges for proteins using Gasteiger charges and to generate the docking input files. AutoDock Vina software was employed for all the protein-ligand calculations and they were applied to the whole protein target (*blind docking*). For hAChE, the grid box was built with a resolution of 1 Å and 60 × 60 × 72 Å and it was positioned at the middle of the protein (x = 116.546; y = 110.33; z = −134.181). For hBuChE, a grid box of 66 × 60 × 74 with grid points separated 1 Å was positioned in the middle of the protein (x = 136.0; y = 123.59; z = 38.56)**.** The resultant structure files were analyzed using Discovery Studio.

### 3.7. Neuroprotection Studies in Human Neuroblastoma Cells

Human SH-SY5Y cells were maintained in a 1:1 mixture of Nutrient Mixture F-12 and Eagle´s minimum essential medium (EMEM) supplemented with 15 nonessential amino acids, 1 mM sodium pyruvate, 10% heat-inactivated FBS, 100 units/mL penicillin, and 100 μg/mL streptomycin. Cultures were seeded into flasks containing supplemented medium and maintained at 37 °C in a humidified atmosphere of 5% CO_2_ and 95% air. For assays, SH-SY5Y cells were sub-cultured in 96-well plates at a seeding density of 5 × 10^4^ cells per well for 2 days. The cells were then incubated with 30 μM rotenone and 10 μM oligomycin-A (R/O) or okadaic acid (20 nM) (Sigma-Aldrich, Madrid, Spain) according to previous studies [18,19] with or without **QT78** at different concentrations for 24 h. In this study, all cells were used at a low passage number (<14).

### 3.8. Measurement of Cell Viability

MTT reduction was performed as described [24] for both HepG2 and SH-SY5Y human cell lines. Briefly, 50 µL of the MTT labeling reagent, at a final concentration of 0.5 mg/mL, was added. After incubation for 2 h, in a humidified incubator at 37 °C with 5% CO_2_ and 95% air (*v*/*v*), the supernatant was removed, the obtained purple formazan product was re-suspended in 100 μL of DMSO. Colorimetric determination of MTT reduction was measured in an ELISA microplate reader at 540 nm. Non-treated cells were considered controls and were taken as 100% viability. 

### 3.9. Statistical Analysis

Statistical analyses were performed using GraphPad Prism software (version 6, San Diego, CA, USA). The data were analyzed by *t*-test or ANOVA with Tukey’s multiple comparison test, as indicated. *P* values less than 0.05 were considered significant.

## 4. Conclusions

In the search for a new MTSM [5] for AD [25,26,27], the most common neurodegenerative disorder [28], the design, synthesis and pharmacological properties of **QT78**, the parent compound of a new family of tacrine derivatives bearing the quinoxaline heterocyclic ring core, was investigated. As a result, we have identified **QT78** as a promising hit compound, which is less toxic than tacrine at high concentrations (from 100 µM to 1 mM), but less potent than tacrine as an inhibitor of both ChEs, with a modest selectivity towards BuChE. At 0.1 and 0.3 μM, **QT78** exhibited a significant neuroprotective effect against different toxic stimuli, including ROS generation and hyperphosphorylation of tau protein. In addition, computational analyses shed light into the toxicological profile, binding and ADME properties of **QT78**. Taken together, the reported data are relevant to the search for new anti-AD agents. Our work is now in progress to prepare and evaluate new, related QTs based on **QT78**. The results will be reported elsewhere in due course.

## Figures and Tables

**Table 1:** In vitro hepatotoxicity of tacrine and **QT78** in HepG2 cells.

Compounds	Viability (%) HepG2 Cells						
	1 µM	3 µM	10 µM	30 µM	100 µM	300 µM	1000 µM
QT78	96.7 ± 2.14 ^ns^	97.6 ± 2.20 ^ns^	97.1 ± 1.64 ^ns^	95.1 ± 1.50 ^ns^	90.5 ± 1.86 ^ns^	78.1 ± 0.94 ***	68.3 ± 2.92 ***
Tacrine	93.4 ± 4.69 ^ns^	90 ± 2.95 ^ns^	88.7 ± 3.42 ^ns^	81.6 ± 4.88 *	64.3 ± 4.54 ***	40 ± 2.20 ***	34.4 ± 2.73 ***

Cell viability was measured as MTT reduction and data were normalized as % control. Data are expressed as the means ± S.E.M. in triplicate of at least three different cell batches. All compounds were assayed at increasing concentrations (1–1000 μM). *** *P* < 0.001, * *P* < 0.05 and ns not significant, with respect to control group. Comparisons between drugs and control group were performed by one-way ANOVA followed by the Newman–Keuls post-hoc test.

## Supplementary Materials

Supporting Information is available free of charge on the website and includes Materials and Methods, NMR and HRMS spectra, and the ADME analysis.
