# Supplementary material for: QuinoxalineTacrine QT78, a Cholinesterase Inhibitor as a Potential Ligand for Alzheimer’s Disease Therapy
Source: Molecules. 2019 Apr 17;24(8):1503. doi: 10.3390/molecules24081503 (PMC6514705; doi:10.3390/molecules24081503)
Supplement: Supplementary file 1 [file molecules-24-01503-s001.zip › molecules-458412-SI.pdf]

Supporting Information

QuinoxalineTacrine QT78, a Cholinesterase Inhibitor as a Potential Ligand for Alzheimer's Disease Therapy

Eva Ramos [1], Alejandra Palomino-Antolín [2,3,4], Manuela Bartolini [5], Isabel Iriepa [6], Ignacio Moraleda [6], Daniel Diez-Iriepa [6,7], Abdelouahid Samadi [7,8], Carol V. Cortina [7], Mourad Chioua [7], Javier Egea [2,3,4,*], Alejandro Romero [1,*] and José Marco-Contelles [7,*]

1 Department of Pharmacology and Toxicology, Faculty of Veterinary Medicine, Complutense University of Madrid, 28040 Madrid, Spain; eva.ramos@ucm.es

2 Molecular Neuroinflammation and Neuronal Plasticity Laboratory, Research Unit, Hospital Universitario Santa Cristina, 28009 Madrid, Spain; apantolin@gmail.com

3 Instituto de Investigación Sanitaria, Hospital Universitario de la Princesa, 28006 Madrid, Spain

4 Instituto-Fundación Teófilo Hernando, Departamento de Farmacología y Terapéutica, Universidad Autónoma de Madrid, 28029 Madrid, Spain

5 Department of Pharmacy and Biotechnology, Alma Mater Studiorum University of Bologna, Via Belmeloro 6, 40126 Bologna, Italy; manuela.bartolini3@unibo.it

6 Departamento de Química Orgánica and Química Inorgánica. Ctra. Madrid-Barcelona, Km. 33,6. Universidad de Alcalá, 28871 Madrid, Spain; isabel.iriepa@uah.es (I.I.); ignacio.moraleda@uah.es (I.M.); daniel.diezi@edu.uah.es (D.D.-I.)

7 Laboratory of Medicinal Chemistry (IQOG, CSIC), C/Juan de la Cierva 3, 28006 Madrid, Spain; samadi@uaeu.ac.ae (A.S.); cvcortina@gmail.com (C.V.C.); mchioua@gmail.com (M.C.)

8 Department of Chemistry, College of Science, United Arab Emirates University, 15551 Al Ain, UAE

* Correspondence: javier.egea@inv.uam.es (J.E.); manarome@ucm.es (A.R.); iqoc21@iqog.csic.es (J.M.-C.); Tel.: +34915574402 (J.E.); +34913943970 (A.R.); +34915622900 (J.M.-C.)

Contents

1. NMR and HRMS spectra of compound QT78…………………………….…...S2-S6

2. ADME analysis…………………………………………………………….S7

3. References……………………………………………………………….S8

1. NMR and HRMS spectra of compound QT78

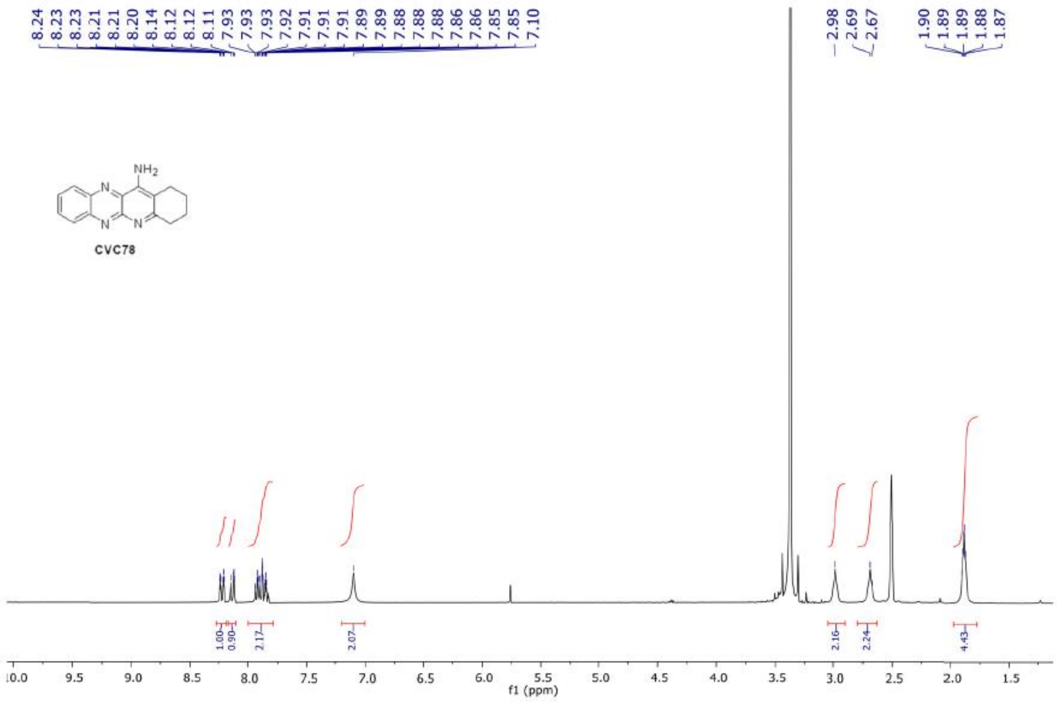

^{1}H-NMR (300 MHz, DMSO-d_6) spectra of **CVC78**

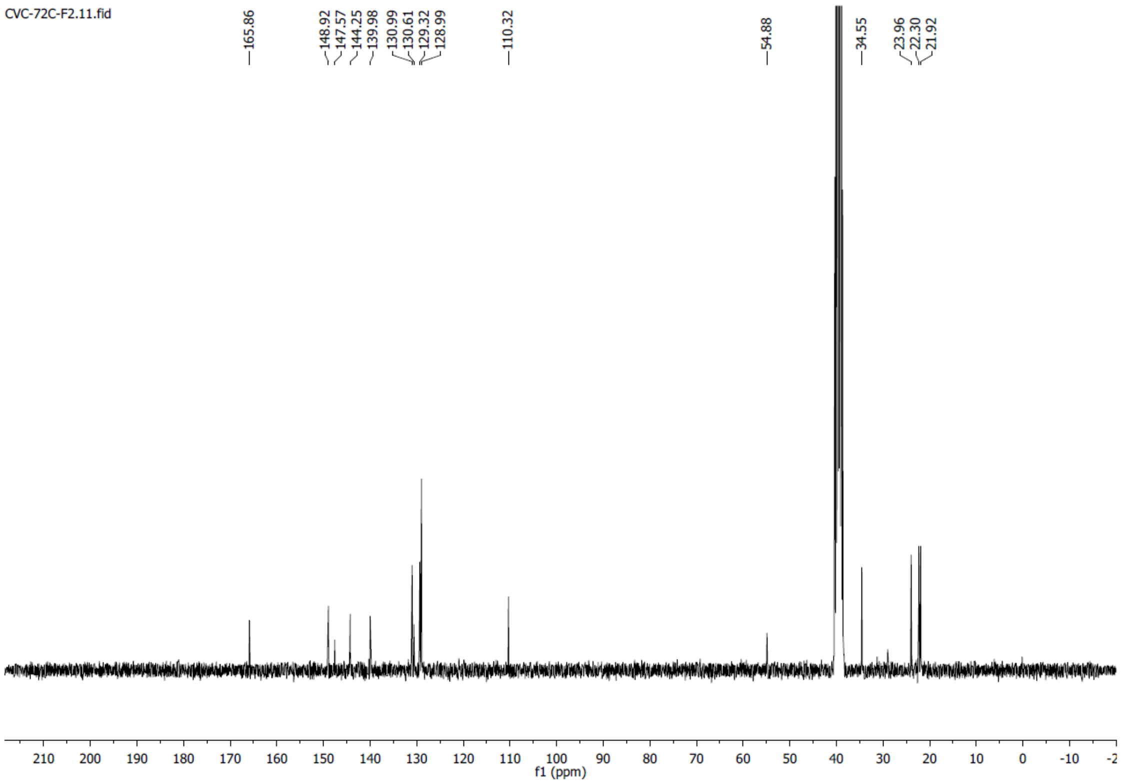
CVC-72C-F2.11.fid
165.86
148.92
147.57
144.25
139.98
130.99
130.61
129.32
128.99
110.32
54.88
34.55
23.96
22.30
21.92
f1 (ppm)

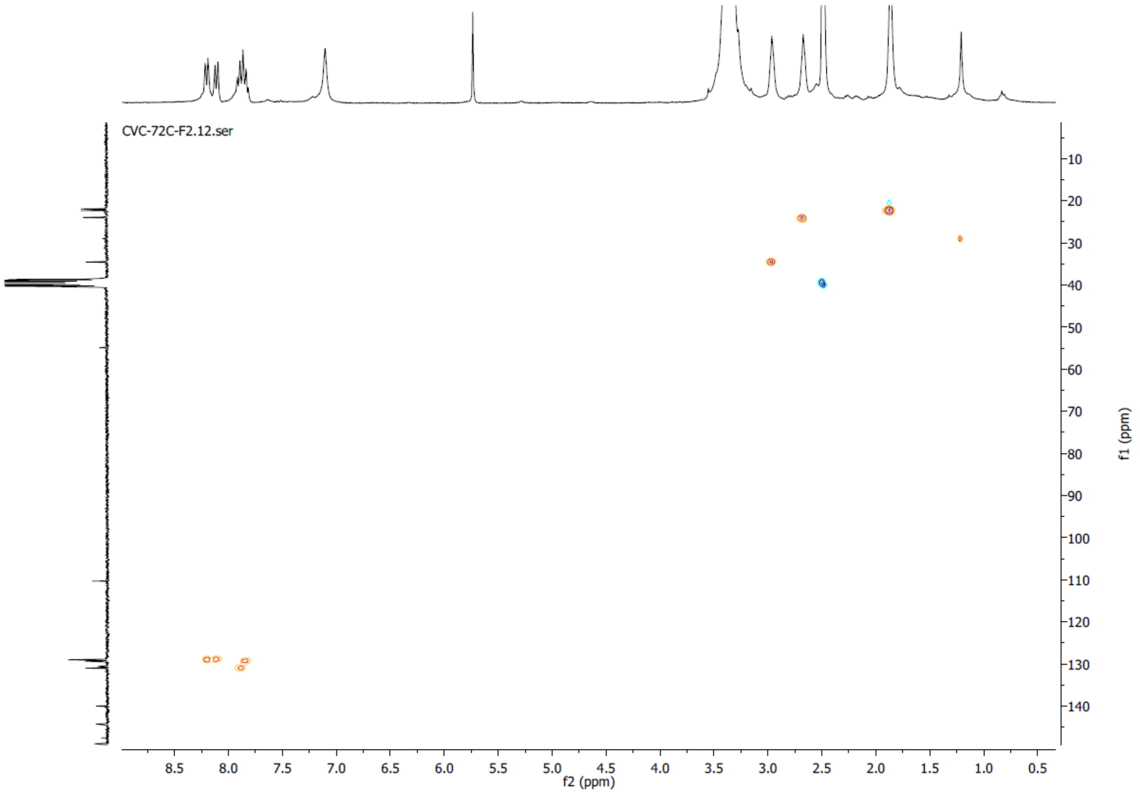
CVC-72C-F2.12.ser
f1 (ppm)
f2 (ppm)

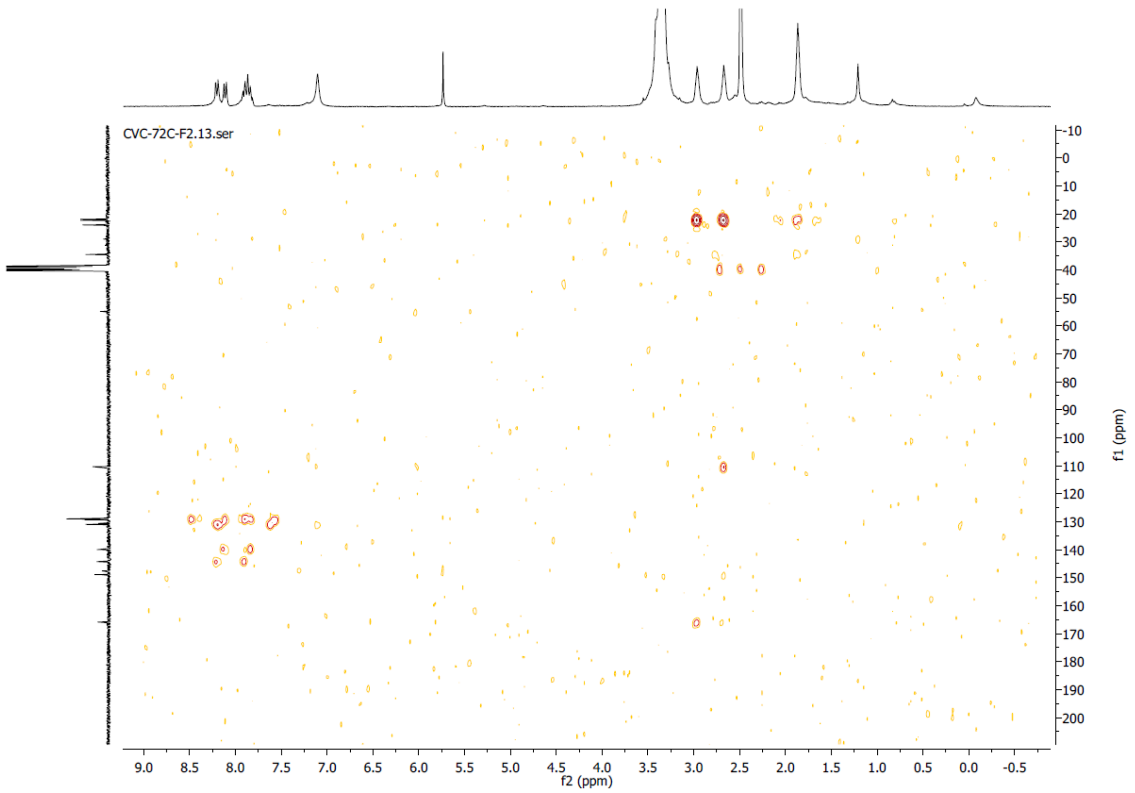
CVC-72C-F2.13.ser
f2 (ppm)
f1 (ppm)

Qualitative Compound Report

**Data File**	12719_j_616_01_.d	**Sample Name**	ACN
**Sample Type**	Sample	**Position**	Vial 21
**Instrument Name**	Instrument 1	**User Name**	
**Acq Method**	ESI_ACN_75_blanco_pos.m	**IRM Calibration Status**	Success
**DA Method**	Defecto_modificado_CS.m	**Comment**	

Compound Table

Compound Label	RT	Mass	Abund	Formula	Tgt Mass	Diff (ppm)
Cpd 1: C15 H14 N4	0.233	250.12261	102881	C15 H14 N4	250.12185	3.05

Compound Label	RT	Algorithm	Mass
Cpd 1: C15 H14 N4	0.233	Find By Formula	250.12261

MS Zoomed Spectrum

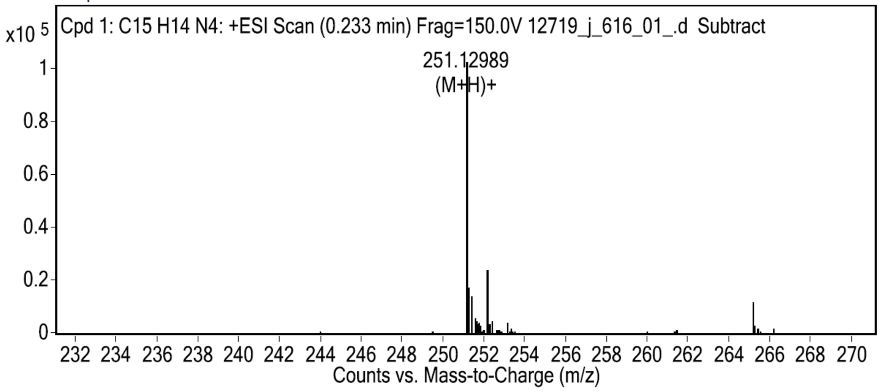

MS Spectrum Peak List

m/z	*Calc m/z*	Diff(ppm)	Abund	Formula	Ion
251.12989	251.12912	3.04	102881	C15 H15 N4	(M+H)+
251.21499			17751		
251.3814			14226		
251.56586			6196		
251.61001			5064		
251.66713			3734		
251.70162			4502		
252.13256	252.13199	2.27	24556	C15 H15 N4	(M+H)+
252.36447			5159		
253.13567	253.13482	3.36	4489	C15 H15 N4	(M+H)+

--- End Of Report ---

Qualitative Analysis Report

**Data Filename**	12719_j_616_01.d	**Sample Name**	j_616
**Sample Type**	Sample	**Position**	Vial 1
**Instrument Name**	Instrument 1	**User Name**	
**Acq Method**	ESI_ACN_75_pos.m	**IRM Calibration Status**	Success
**DA Method**	Defecto_modificado_CS.m	**Comment**	

User Spectra

Fragmentor Voltage	Collision Energy	Ionization Mode
150	0	Esi

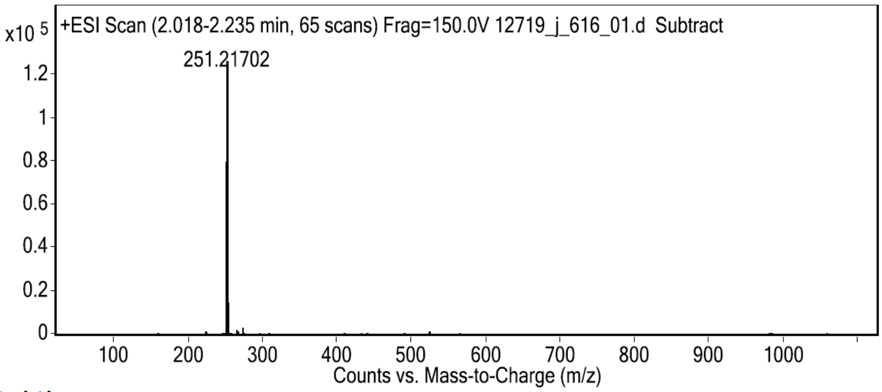

Peak List

m/z	z	Abund
251.13420	2	79917
251.21702	2	126216
251.35296	2	48824
251.36351		55743
251.54555		20138
251.60801		16216
251.64293	2	26188
252.13293	2	114953
252.22077	2	20875
253.13503		15287

--- End Of Report ---

2. ADME analysis

Table S1. Physicochemical properties for compound **QT78** calculated using Qikprop

Molecule	MW	SASA	Volume	donorHB	accptHB	QPlogPo/w	QPlogS	QPPCaco	PSA	QPlogBB	metab	QPlogKhsa	% HOA	ROF
**QT78**	250.302	250.302	834.576	1.500	4.000	2.534	-3.746	1284.587	59.549	-0.332	3	0.079	100.000	0

MW: Molecular weight of the molecule (130.0-725.0). SASA: Total Solvent Accessible Surface Area, in square angstroms, using a probe with a 1.4 Å radius (limits 300.0-1000.0). volume: Total solvent-accessible volume, in cubic angstroms, using a probe with a 1.4 Å radius (limits 500.0-2000.0). donorHB: Estimated number of hydrogen bonds that would be accepted by the solute (limits: 2.0-20.0). accptHB: Estimated number of hydrogen bonds that would be donated by the solute (limits: 0.0-6.0). QPlogPo/w: Predicted octanol/water partition coefficient (limits -2.0-6.5). QPlogS: Predicted aqueous solubility. S, in mol/dm^3, is the concentration of the solute's saturated solution that is in equilibrium with crystalline solid (limits -6.5-0.5). QPPCaco: Predicted apparent Caco-2 cell permeability in nm/sec. Caco-2 cells is a model for the gut-blood barrier. QikProp predictions are for non-active transport. (< 25 poor, > 500 great). PSA: Van der Waals surface area of polar nitrogen and oxygen atoms (limits 7.0-200.0). QPlog BB: Predicted brain/blood partition coefficient (limits -3.0–1.2). metab: Number of likely metabolic reactions (limits 1-8). QPlogKhsa: Prediction of binding to human serum albumin (limits -1.5-1.5). HOA: Predicted qualitative Human Oral Absorption on 0 to 100% scale. ROF: Number of violations of Lipinski's Rule of Five [1] (molecular weight < 500, QPlogPo/w < 5, number of hydrogen bond donor ≤ 5, number of hydrogen bond acceptors HB ≤ 10). ROT: Number of violations of Jorgensen's rule of three [2,3] (QPlogS> -5.7, QPCaco> 22 nm/s, number of primary metabolites < 7).

3. References

1. Lipinski, C. A.; Lombardo, F.; Dominy, B. W.; Feeney, P. J. Experimental and computational approaches to estimate solubility and permeability in drug discovery and development settings. *Adv. Drug. Deliv. Rev.* **2001**, *46*, 3-26.
2. Jorgensen, W. L.; Duffy, E. M. Prediction of drug solubility from Monte Carlo simulations. *Bioorg. Med. Chem. Lett.* **2000**, *10*, 1155-1158.
3. Duffy, M. E.; Jorgensen, W. L. Prediction of Properties from Simulations: Free energies of solvation in hexadecane, Octanol, and Water. *J Am Chem Soc.* **2000**, *122*, 2878-2888.
